# Generation of Myeloid Cells in Cancer: The Spleen Matters

**DOI:** 10.3389/fimmu.2020.01126

**Published:** 2020-06-05

**Authors:** Chong Wu, Qiaomin Hua, Limin Zheng

**Affiliations:** MOE Key Laboratory of Gene Function and Regulation, State Key Laboratory of Biocontrol, School of Life Sciences, Sun Yat-sen University, Guangzhou, China

**Keywords:** cancer, myeloid cell, spleen, hematopoietic stem/progenitor cell, myelopoiesis

## Abstract

Myeloid cells are key components of the tumor microenvironment and critical regulators of disease progression. These innate immune cells are usually short-lived and require constant replenishment. Emerging evidence indicates that tumors alter the host hematopoietic system and induce the biased differentiation of myeloid cells to tip the balance of the systemic immune activities toward tumor-promoting functions. Altered myelopoiesis is not restricted to the bone marrow and also occurs in extramedullary organs. In this review, we outline the recent advances in the field of cancer-associated myelopoiesis, with a focus on the spleen, the major site of extramedullary hematopoiesis in the cancer setting. We discuss the functional specialization, distinct mechanisms, and clinical relevance of cancer-associated myeloid cell generation from early progenitors in the spleen and its potential as a novel therapeutic target.

## Introduction

Cancer is now viewed as an ecological disease in which interactions between neoplastic, stromal, and infiltrating immune cells profoundly regulate disease progression. Myeloid cells are major components of this ecosystem. These cells belong to the innate immune system and comprise various mononuclear and polymorphonuclear phagocytes and precursors, including monocytes/macrophages (Mos/Mϕs), dendritic cells (DCs), granulocytes, and myeloid-derived suppressor cells (MDSCs). Over the past two decades, a wealth of studies has revealed the crucial roles that myeloid cells play in many, if not all, steps of tumor initiation, progression and metastasis (1–6). The importance of myeloid cells has been further underlined by identifying the broad involvement of myeloid cells in regulating treatment responses and has thereby spurred interest in therapeutically targeting these cells (7–12).

In addition to directly modulating myeloid cells in tumor tissues using small molecules (13–16), antibodies (17–19), and nanoparticles (20–24), a novel myeloid cell-targeting strategy is now emerging into the research spotlight. The idea is to limit the tumor-supporting myeloid cell response at its root by restraining tumor-associated myelopoiesis. Tumor progression often parallels a coordinated expansion and continuous accumulation of myeloid cells such as tumor-associated macrophages (TAMs) (11, 25–28), neutrophils (TANs) (5, 15, 29–31), and MDSCs (3, 32, 33). Considering that cells of the myeloid compartment are generally short-lived, this growing and fast-turnover pool of tumor-associated myeloid cells needs to be promptly and constantly regenerated from hematopoietic stem and progenitor cells (HSCs and HPCs, or HSPCs combined). Therefore, tumors interfere with host hematopoiesis and skew the process toward the generation of myeloid cells with tumor-promoting properties. The generality and importance of hematopoietic deviation in cancers are supported by evidence from both human and mouse studies (34–38). Notably, hematopoietic alteration is not restricted to the bone marrow (BM), the primary hematopoiesis site for adults, but has also been observed in multiple extramedullary organs. However, our knowledge about the nature and properties of cancer-induced myelopoiesis, in particular the necessity and advantages of extramedullary hematopoiesis, is still limited. In this review, we briefly introduce myelopoiesis in different sites discovered to date in the context of solid tumors and then focus on the spleen, the major site of extramedullary myelopoiesis. The expansion of downstream immature [e.g., MDSCs (2, 39, 40)] or mature myeloid cells [e.g., TAMs (41)] has been well-summarized in several recent reviews; thus, here, we focus on the role of early HSPCs in cancer-associated myelopoiesis.

## Sites of Myelopoiesis in Cancers

At steady state, HSPCs reside primarily in the BM and generate cells of the blood and immune systems (42, 43), with a small subpopulation constantly recirculating between the BM and blood (44, 45). These peripheral HSPCs survey extramedullary tissues and respond rapidly to danger signals to resolve hematopoietic/immunological stress conditions (46). In recent years, the paradigm that HSPCs divide in response to peripheral cytopenia has given way to one in which HSPCs can sense environmental stimuli and pro-inflammatory cytokines directly and thus can actively serve as a foundation for the immune response (47, 48). These mechanisms operating in “emergency” myelopoiesis are hijacked by cancers, which instruct HSPC activity, at least in part, through the constant and progressive release of cytokines, chemokines, and metabolites (49, 50). Here, we summarize the recent discoveries in cancer-associated myeloid cell generation taking place in the BM and extramedullary sites.

### Bone Marrow

In the BM, the binding of stromal-cell-derived factor-1 (SDF-1, also known as CXCL12) to its receptor CXCR4 represents a critical axis in the BM retention and homing of HSPCs (45, 51–53). Granulocyte colony-stimulating factor (G-CSF) is known to antagonize this SDF-1/CXCR4 axis, modulate BM HSPC mobilization, and direct hematopoietic differentiation. A recent study using a mouse model of breast cancer showed that tumor-derived G-CSF induces the expansion and differentiation of HSPCs to skew hematopoiesis toward the myeloid lineage. Myeloid-biased hematopoiesis results in the systemic expansion of myeloid suppressors with the distinguishing characteristics of tumor-induced immunosuppressive neutrophils (36). These results are consistent with previous findings showing that the BM CD11b^+^Gr1^+^ myeloid cell compartment expands in response to tumor-derived G-CSF and is functionally altered before these cells are mobilized into the circulation (54, 55), via the activation of the retinoic-acid-related orphan receptor (RORC1/RORγ) and CCAAT/enhancer-binding protein β (C/EBPβ) pathways (56).

In addition, other hematopoietic cytokines, such as macrophage colony-stimulating factor (M-CSF) (57), granulocyte/macrophage colony-stimulating factor (GM-CSF) (58), vascular endothelial growth factor A (VEGF-A) (59, 60), placental growth factor (PlGF) (59, 61), osteopontin (62, 63), transforming growth factor-β (TGF-β) (60), and tumor necrosis factor-α (TNF-α) (60, 64), are known to influence hematopoiesis and are secreted by a variety of solid cancers to affect the BM (65). Although the precise effect and mechanisms are not yet fully elucidated, these cytokines may also impact the differentiation pattern of HSPCs and regulate tumor-promoting myeloid cell responses.

### Primary Tumor and Pre-metastatic Sites

In the context of cancer, we have found that circulating HSPCs from patients with various types of solid tumor, including hepatocellular, breast, cervical, esophageal, gastrointestinal, lung, and ovarian tumors, exhibit a generalized myeloid bias that skews toward granulocytic differentiation (35). Whether these trafficking HSPCs have a preset destination other than returning to the BM remains unclear. One possible extramedullary site for HSPC residence and function is the tumor. BM-derived HSPCs have been observed within the stroma of primary tumors and are thought to promote tumor progression (59, 62, 63, 66). In support of these findings, we have found that there is significant infiltration of CD133-expressing precursor cells with multipotent colony-formation capabilities in human colon cancer tissues (35, 67). These HSPCs give rise to immature myeloid cells with a potent immunosuppressive function in a glutamine metabolism-dependent manner (67). Recent studies have demonstrated that in addition to homing to the primary tumor, a distinct subset of HSPCs that express vascular endothelial growth factor receptor 1 (VEGFR1; also known as Flt1) can home to tumor-specific pre-metastatic sites. These HSPCs express necessary adhesion molecules and growth factors and differentiate into immunosuppressive MDSCs to form a permissive niche for incoming tumor cells (61, 68, 69).

In contrast to the above findings, there are some reports based on transplant-treatment models showing that the transfer of BM-derived HSPCs can enhance adoptive T cell immunotherapy (ACT) in mouse melanoma (70) and glioma models (71), thus arguing that HSPCs can play an antitumor role in ACT. Wildes et al. reported that the combination of ACT and HSPC transfer could lead to HSPC differentiation into immune-stimulating DCs in mouse glioma. The treatment began with a sublethal- or lethal-dose total body irradiation, followed by adoptive transfer of autologous HSPCs and tumor-reactive T cells. These T cells released IFN-γ in the brain tumor microenvironment to augment HSPC differentiation into potent DCs, which in turn further activated tumor-reactive cytotoxic T lymphocytes (CTLs) in a positive feedback manner (72). Such treatments, involving total body irradiation, may raise concerns regarding the translational value, but these studies did provide hints of the potential mechanisms by which altering the tumor microenvironment/hematopoietic niche may reprogram the typical immunosuppressive myelopoiesis and function of HSPCs. Thus, current evidence suggests that the existence, biological nature, and clinical relevance of myelopoiesis in primary tumors and pre-metastatic sites are highly heterogeneous and tumor-dependent.

### Spleen

The spleen is now viewed as the prominent site of extramedullary hematopoiesis (EMH) in cancers. The spleen, which is located in the abdominal cavity, right beneath the diaphragm and connected to the stomach, is the largest secondary lymphoid organ in the body. The spleen plays a crucial role in filtering antigenic particles and abnormal cells from the blood, destroying aged erythrocytes, and recycling iron and is an important organ for the differentiation and activation of T and B cells and production of antibodies (73–75). In hematology, the spleen serves as an important reservoir of monocytes (76, 77), platelets (78, 79), and memory B cells (80). The spleen is also a significant site of hematopoiesis throughout vertebrate evolution and during fetal development in humans (81). Although the contribution of splenic EMH in steady-state adults seems trivial, a vast spectrum of hematopoietic stresses, including myelofibrosis (82), anemia (83), pregnancy (84), infection (85, 86), myeloablation (87), myocardial infarction (88, 89), diabetes (90), atherosclerosis (91, 92), colitis (93), and spondyloarthritis (94), can induce profound EMH in the spleen. Splenic EMH also occurs in the context of cancer. In addition to reports on the expansion of myeloid precursors in the spleen (95–97), Cortez-Retamozo et al. found that the spleen of hosts bearing lung adenocarcinomas accommodates a large number of HSPCs, including HSCs and granulocyte/macrophage progenitors (GMPs), that are phenotypically and functionally analogous to their BM counterparts. These splenic HSPCs give rise to myeloid descendants, such as monocytes and neutrophils, that subsequently migrate to the tumor and exert tumor-promoting functions (34, 98). Consistently, in various mouse models with transplanted, genetically engineered, or chemically induced malignancies and in patients with hepatocellular, gastric, renal, or pancreatic cancers, the spleen accommodates a profound expansion of early HSPCs and supports myeloid-biased myelopoiesis, suggesting the generality of splenic myelopoiesis in various types of solid tumors (37). It is also noteworthy that cancer-induced EMH does not produce only myeloid cells; in late-stage cancers, the spleen generates unique erythrocytic cell populations to further alleviate the disease (99–101).

To evaluate the significance of splenic myelopoiesis in cancers, two central questions need to be addressed: (1) What is the relative contribution of splenic myelopoiesis, compared with that of the BM and other extramedullary tissues, to cancer-associated myeloid cells? (2) Is splenic myelopoiesis a mere complement to BM hematopoiesis or does it play a unique role in generating particular myeloid subsets? To date, the relative contribution of splenic myelopoiesis is controversial. Current evidence suggests that this depends on the type of cancer and the settings of the tumor model. In some experiments, splenectomy causes a significant decrease in the tumor-infiltrating myeloid population and restricts tumor growth (34, 102–104), whereas in other settings, these effects seem marginal (37, 38, 105). Beyond the comparison of production capacity, we recently found that although splenectomy does not change the frequency or distribution of tumor myeloid cells in a hepatoma model, the abrogation of splenic EMH reduces the expression of arginase 1 (Arg1) and abolishes the suppressive activity of tumor CD11b^+^Ly6G^+^Ly6C^low^ granulocytic MDSCs, the major MDSC subset in that tumor (37). Thus, emerging studies suggest that splenic myelopoiesis is more than a complement to BM myelopoiesis and may represent myeloid cell biogenesis that is functionally and mechanistically different from its BM counterpart. This mechanism is important for systemic tumor-promoting myeloid cell responses. Therefore, a systematic understanding of cancer-induced splenic EMH (myelopoiesis) is critical for guiding the development of novel therapeutic strategies targeting myeloid cell responses.

### Other Extramedullary Organs

Hematopoiesis can take place in many tissues (106–108). Although EMH plays a physiological role during fetal development, its occurrence after birth is typically abnormal, usually associated with inflammation or hematological diseases such as myelofibrosis, leukemia, and hemolytic anemia. In cases of malignant solid tumors, this process seems to rarely develop in organs other than the spleen. The liver is an important hematopoietic organ during the fetal stage, but liver hematopoiesis in solid cancers has only been reported in patients undergoing liver transplantation (109, 110). Even in the context of hepatoma, there is no detectable HSPC accumulation in the non-cancerous livers of mice bearing orthotopic hepatic tumors or in the tumor stroma of patients with hepatocellular carcinoma (37). Similarly, a recent study revealed that the lung is a reservoir for HSPCs and an important site of platelet biogenesis in adults (111). However, reports on lung hematopoiesis in cancers are still rare (112).

## Mechanisms Regulating Splenic Myelopoiesis

**Figure 1:**
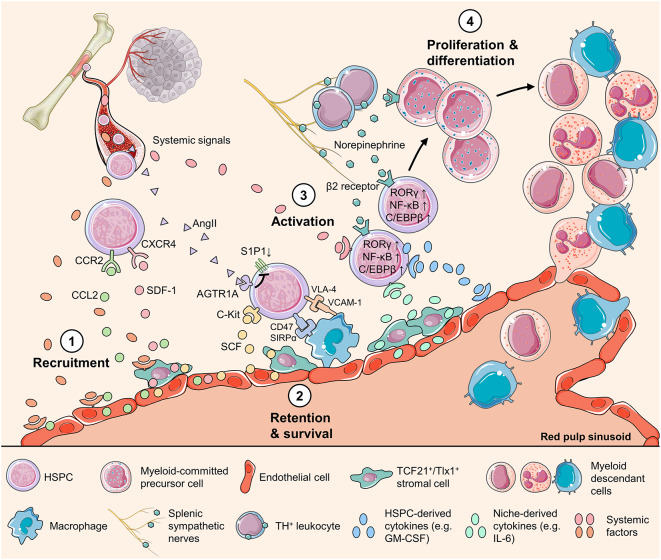
Mechanisms regulating HSPC activity in the spleen. Schematic representation of the HSPC behavior during splenic myelopoiesis, showing multiple cell types and factors of various origins that directly or indirectly regulate HSPC activity. The splenic HSPC response is initiated with (1) increased production of chemokines, such as SDF-1 and CCL2, by endothelial cells and stromal cells around sinusoids. This change of chemokine production might be triggered by systemic factors that convey organismal stress messages. (2) HSPC survival is supported with the key niche-derived cytokine SCF and HSPCs express CD47 to avoid being engulfed by splenic macrophages. In addition, HSPCs express VLA-4 and downregulate S1P1 to maintain in the splenic niche. (3) Activated by systemic, niche-derived, and neural signals, splenic HSPCs upregulate transcription factors including RORC1/RORγ and C/EBPβ to direct myeloid-biased differentiation. Emerging evidence highlights the roles of the HSPC endogenous cytokines such as GM-CSF, and the transcription factor NF-κB that drives the production of cytokines in HSPC, as key regulators of HSPC behavior. (4) HSPCs proliferate and differentiate into different myeloid cell populations to respond to the body's or, unfortunately, the tumor's call. AGTE1A, type1A angiotensin II receptor; AngII, angiotensin II; C/EBPβ, CCAAT/enhancer-binding protein β; CCL2, C-C motif chemokine ligand 2; CCR2, C-C motif chemokine receptor 5; CXCR4, C-X-C motif chemokine receptor 4; NF-κB, nuclear factor kappa-light-chain-enhancer of activated B cells; RORγ, related orphan receptor γ; S1P1, sphingosine-1-phosphate receptor 1; SCF, stem cell factor; SDF-1, stromal-cell-derived factor-1; SIRPα, signal regulatory protein α; Tlx1, T-cell leukemia homeobox protein 1; VCAM-1, vascular cell adhesion molecule-1; VLA4, very late antigen-4.

Splenic EMH is a highly flexible and adaptable response that differs in scale and output in homeostasis, under physiological stress conditions, and in various disease states. How splenic HSPC activity and the EMH niche are shaped to adapt to the organismal environment is incompletely understood, but it may involve at least two essential mechanisms: the selective recruitment of HSPCs and dynamic HSPC-niche interactions (Figure 1). Below, we discuss the potential mechanisms by which splenic EMH is induced and regulated in the context of cancer.

### Stromal and Endothelial Cells

The structure and fundamental functions of the spleen have been thoroughly described in recent reviews (73–75). The spleen is organized in regions called the red pulp and white pulp. During EMH, HSCs are found mainly around sinusoids in the red pulp. Stem cell factor (SCF, also known as kit ligand) and SDF-1 are key factors in the BM niche of HSCs (42, 51–53). Based on the similarities between splenic EMH and normal BM hematopoiesis under physiological stress conditions such as myeloablation, blood loss, and pregnancy, the splenic EMH niche components are thought to be analogous to those in the BM. Indeed, murine splenic stromal cells (PDGFRβ^+^TCF21^+^ and Tlx1^+^) and endothelial cells have been found to be the major source of SCF, whereas a fraction of the non-endothelial SCF-expressing stromal cells are the source of SDF-1. EMH induction significantly expands the SCF-expressing endothelial and stromal cell populations to which most splenic HSPCs are found to be adjacent (113, 114). However, it should be noted that the structure of the human spleen is different from that in mice in many aspects (74, 115), and this may also be true regarding the EMH niche components. For example, SDF-1 expression has been detected in humans (116) but not mouse (113) splenic endothelial cells. A detailed depiction of the EMH niche in the human spleen is still lacking.

Although the splenic EMH niche is poorly understood, growing evidence indicates that tumor-induced splenic EMH may not entirely mimic BM EMH. In hepatoma-bearing mice, SDF-1 expression in the spleen is markedly decreased, rather than increased, at both the RNA and protein levels. In contrast, the CCR2 ligand CCL2, mainly expressed by VE-cadherin^+^ stromal/endothelial cells, has been found to profoundly increase as tumor grows (37). CCR2 is expressed on a subset of the highly active HSPC population in the circulation. Peripheral CCR2^+^ HSPCs are armed with pattern recognition receptors (PRRs) such as TLR4 and TLR2 and preferentially differentiate into reparative myeloid cells, such as M2 macrophages, representing the most upstream point of increased local myelopoiesis after aseptic inflammation, liver injury, and myocardial infarction (117, 118). The CCL2/CCR2 axis is employed to mediate the splenic recruitment of HSPCs in tumor-bearing mice. A lack of CCR2 expression on HSPCs reduces splenic myelopoiesis, impairs the suppression activity of tumor MDSCs, allows an increase in the number of tumor-infiltrating IFNγ^+^CD3^+^CD8^+^ CTLs, and enhances immunotherapy efficacy (37, 38). Thus, this selective recruitment mechanism may in part account for splenic immunosuppressive myelopoiesis in cancer.

### Endogenous HSPC Signals and the HSPC-Niche Interplay

In addition to niche signaling, it is well-accepted that HSPCs themselves can secrete a long list of cytokines that modulate their own function in an autocrine or paracrine manner in response to stimuli (86). Although the contribution of these endogenous signals to the inflammatory response in inflamed tissues remains doubtful, HSPC-derived pro-inflammatory factors may play a significant role in the hematopoietic niche. We recently identified a subset of GM-CSF-expressing HSPCs found exclusively in the spleens of mice bearing different types of solid tumors but not in the BM, control mouse spleen, or spleens of mice with EMH induced by repeated bleeding (37). GM-CSF, as shown in other studies (93, 94, 119), can direct HSPC proliferation and myeloid differentiation. More surprisingly, GM-CSF-expressing splenic HSPCs, but not BM HSPCs, can readily generate myeloid suppressors independent of the presence of tumors when transferred into tumor-free mice (37). These findings represent the tip of a far larger iceberg. It is logical to assume that under pathological conditions, a considerable proportion of HSPCs may produce a broad spectrum of cytokines in the splenic niche to direct splenic EMH. Moreover, one may infer that these cytokines would also affect the dynamic niche. If so, the HSPC-niche cell interplay would be reciprocal. Understanding how the unique combination of HSPC-derived and niche factors orchestrate HSPC activity to regulate the output in the spleen of a tumor-bearing host will certainly advance our understanding of cancer-induced splenic myelopoiesis.

### Macrophages as Double-Edged Swords in Regulating Splenic EMH

Splenic red pulp macrophages also play an important role in regulating splenic EMH. On the one hand, macrophages retain HSPCs in the splenic red pulp by providing adhesion via vascular cell adhesion molecule-1 (VCAM-1) and may thus promote splenic EMH. Hindering macrophage maturation using *in vivo* RNAi silencing, depleting splenic macrophages, or silencing VCAM-1 in macrophages releases HSCs from the spleen and compromises splenic EMH (120). On the other hand, macrophages can regulate splenic EMH by phagocytosing redundant HSPCs in the spleen. According to an early study, the phagocytosis of HSPCs by the numerous active macrophages present in the cords of the red pulp results in limited EMH in human spleens (121), suggesting that phagocytosis is a key mechanism regulating splenic HSPC activity. CD47 is a “don't eat me” signal that inhibits phagocytosis by binding to its receptor signal regulatory protein α (SIRPα), which is expressed on phagocytes. HSPCs upregulate CD47 expression just before and during their migration to the periphery to avoid inappropriate phagocytosis (122). Thus, the downregulation of CD47 expression might lead to the clearance of splenic HSPCs as they age or become dysfunctional. Therefore, macrophages could play dual roles in modulating splenic EMH. However, the roles that splenic macrophages play in regulating cancer-induced splenic EMH during cancer development and the relationship between these functions are still largely unknown. Since therapies targeting macrophages (21, 32, 123) and anti-CD47 treatment (122, 124, 125) are emerging as novel anti-tumor strategies, a deeper understanding of these issues may reveal the impact of these treatments on splenic EMH.

### The Nervous System and Neural Signal-Expressing Cells

Recent studies have revealed an intricate, panicle-shaped sympathetic architecture in the spleen (126). Most detectable nerves entering the spleen arise from the nerve plexus that surrounds branches of the splenic artery and are catecholaminergic (127). Such sympathetic architecture is absent in the other classic lymphoid organs, but whether and how this unique innervation of the spleen contributes to the distinct EMH remains largely unclear. A recent study showed that in liver cancer models, blocking β-adrenergic signaling could prevent the redistribution of splenic myeloid cells and inhibit tumor growth induced by restraint stress (128). In addition, immune cells such as macrophages and T cells can also produce catecholamines (129, 130). Although data from cancer models are limited, in hyperglycemic conditions, the spleens of diabetic patients and mice harbor increased numbers of tyrosine hydroxylase (TH)-expressing leukocytes that produce catecholamines, and GMPs that are actively proliferating. These two events are closely linked, as the interaction of catecholamine and β2 adrenergic receptors expressed on splenic GMPs mediates GMP proliferation and myeloid cell production. Moreover, TH^+^ leukocytes are located close to splenic nerves and express high levels of neuropeptide Y receptors, suggesting that these cells are involved in neuroimmune communication (90). These mechanisms may also exist in cancer-bearing hosts. Future studies are required to identify the roles of the nervous system and neural signal-expressing cells in regulating cancer-induced myelopoiesis.

### Signals From Distant Organs

Although it is almost certain that tumors can profoundly affect splenic myelopoiesis, either directly or indirectly, as the tumor influences the BM (65), the molecular mechanisms remain largely undetermined. In the scenario of cancers expressing high levels of CSFs, these cytokines may be the major cause of HSPC mobilization, splenomegaly, and vigorous splenic myelopoiesis (36, 97, 131, 132). In addition to hematopoietic cytokines, other tumor-derived factors, e.g., peptides and carbohydrates, can also impact on HSPC behaviors. Cortez-Retamozo et al. showed angiotensin II (AngII), a peptide hormone that belongs to the renin-angiotensin system, may also play a significant role in HSPC retention (98). They found that the expression of angiotensinogen, the AngII precursor, was upregulated in a mouse model of lung adenocarcinoma as well as in human lung cancer stroma. AngII could directly induce HSPC amplification in the splenic red pulp, suppressing the signaling between sphingosine-1-phosphate receptor 1 (S1P1) and sphingosine-1-phosphate and thus sequestrating HSPCs in the spleen. A 3 week treatment with the angiotensin-converting enzyme inhibitor enalapril suppressed the expansion of HSPCs in the spleen but not in the BM and reduced the amplification of monocytes in the spleen and macrophage accumulation in the lungs (98). Heparan sulfate proteoglycans (HSPGs) represent another class of potential factors that tumors may exploit to impact on host hematopoiesis. These molecules are composed of a core protein to which chains of the glycosaminoglycan, heparan sulfate (HS), are covalently bound. HSPGs are wildly expressed and released by most types of tumor cells (133) and have known essential effect on furnishing the myelopoiesis microenvironment (134). Early studies have implicated that these structures may play an important role in regulating splenic EMH in tumor conditions (135, 136), but the exact mechanism remains to be further explored and validated. Nevertheless, these potential mechanisms exemplify how the tumor remotely expands the splenic HSPC response and regulates splenic myelopoiesis.

**Figure 2:**
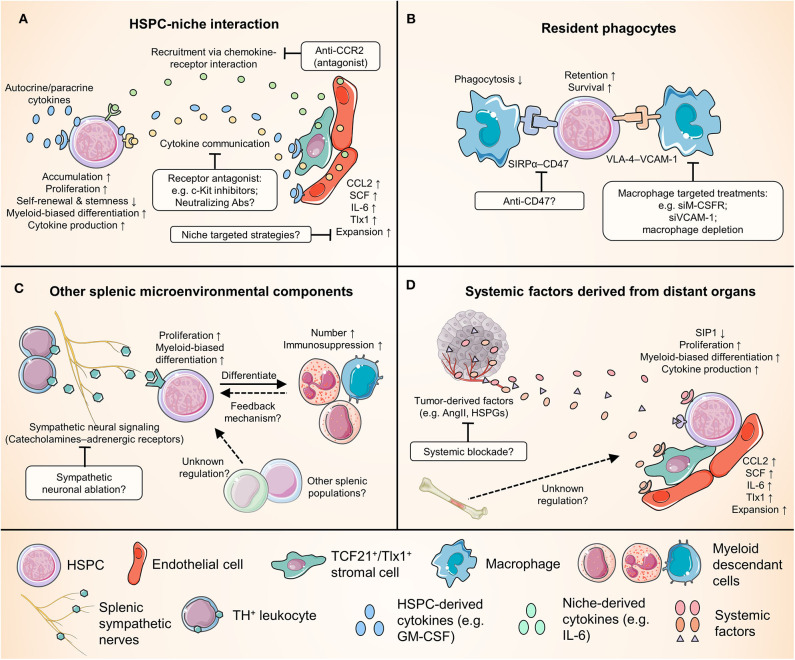
Crucial relationships for splenic HSPCs in cancer and potential therapeutic targets. Numerous cell types and factors come into play in regulating the cancer-induced splenic HSPC activity, providing a wide range of potential therapeutic targets. This figure categorizes these interplays into four groups, and highlights examples of some potential therapies. **(A)** The complex reciprocal interplay between HSPCs and niche cells. **(B)** The interaction between HSPCs and splenic macrophages. Note that macrophages could play dual roles in modulating splenic EMH. **(C)** The regulation of splenic HSPC response by other splenic microenvironmental components, e.g., the sympathetic neurons and leukocytes that produce catecholamines. **(D)** The remote control of splenic myelopoiesis by tumor and possibly other distant organs such as the bone marrow. ACE, angiotensin-converting enzyme; AngII, angiotensin II; CCL2, C-C motif chemokine ligand 2; CCR2, C-C chemokine receptor 2; HSPGs, heparan sulfate proteoglycans; IL-6, interleukin-6; M-CSFR, macrophage colony-stimulating factor receptor; S1P1, sphingosine-1-phosphate receptor 1; SCF, stem cell factor; SIRPα, signal regulatory protein α; Tlx1, T-cell leukemia homeobox protein 1; VCAM-1, vascular cell adhesion molecule-1; VLA4, very late antigen-4.

To date, we have limited information about the mechanism by which tumors systemically modulate the scale, functional characteristics, and output of splenic HSPC responses. Several important questions warrant investigation. For example, do the systemic factors derived from the tumor qualitatively and quantitatively affect splenic EMH and myelopoiesis to the same extent as they impact BM hematopoiesis? In addition, although splenic EMH is myeloid-biased in early stages, cancer-induced EMH also generates unique tumor-promoting cells of the erythrocytic lineage in late-stage cancers (99–101); what tumor-derived signals through which mechanism mediates this functional shift of splenic EMH? A better understanding of these issues is crucial to delineate cancer-associated myelopoiesis and myeloid cell responses and pave the way to developing novel strategies for cancer immunotherapy (Figure 2).

## Clinical Relevance of Splenic Myelopoiesis in Cancer

### Splenic EMH in Humans

Although the role of splenic EMH in tumor-induced myelopoiesis and disease progression is increasingly being appreciated in animal models, it remains largely unknown whether the same is true in cancer patients. Previous studies in human subjects suggested that there is very limited hematopoiesis in the fetal spleen (81, 121) and that adult spleens from individuals without EMH (exemplified by increased circulating HSPC numbers) do not contain committed hematopoietic progenitors (137). Thus, it has been speculated that the human spleen may not function as an EMH site for altered myelopoiesis. However, this view has been challenged by a growing body of more recent data. First, a study using functional identification assays demonstrated that although the frequency of early colony-forming units (CFUs) in the spleen of healthy adults was significantly lower than that in the BM, the frequency of cobblestone area-forming cells in long-term stromal cultures and the frequency of secondary CFUs in long-term culture-initiating cells (both assays determine the long-term HSCs) were comparable in the spleen and BM (138). These results suggest that the human spleen is an important reservoir of dormant early HPCs or even HSCs at steady state. Second, the significant role splenic EMH plays in human pathology is now emerging. The expansion of splenic HSPCs has been observed in patients with osteopetrosis (137), myelofibrosis (139), and acute myocardial infarction (88), supporting the hypothesis that the spleen is the preferred site for extramedullary “emergent” hematopoiesis in a wide spectrum of pathological conditions.

We found that in cancer, in addition to the generalized myeloid bias in the circulating HSPC compartment from various patients with solid tumors, there is a positive correlation between the levels of circulating GMPs and clinical stages in patients with hepatocellular (HCC), cervical and colorectal carcinomas. Moreover, within a small group of HCC patients, Kaplan-Meier analysis revealed that the frequency of GMPs was negatively correlated with the time to progression (35). Accordingly, elevated proportions of HSPCs in the circulation were also found in newly diagnosed cancer patients with rhabdomyosarcoma and breast cancer and correlated with an increased risk for metastatic relapse (69). These data indicate that there is a correlation between heightened EMH and the progression of human cancer. Moreover, the spleen has been reported to be a site of cancer-related EMH in metastatic carcinomas of different origins, including lung, breast, prostate, and kidney (140). We and others have confirmed and extended this observation by showing the splenic accumulation of HSPCs and myeloid cells in patients with different types of solid tumor (34, 37). In a cohort of patients with gastric cancers, the accumulation of HSPCs was inversely correlated with reduced overall survival after surgery (34, 37). However, larger-scale studies are required to confirm the clinical relevance of splenic EMH in cancer and to test the utility of HSPC number and phenotype in circulation as biomarkers to predict disease progression and the therapeutic response in cancer patients.

### Impact of Splenectomy on Malignancy

To date, most clinical data regarding the impact of spleen function on malignancy come from studies on splenectomized patients. These studies relate to issues in two categories: (1) whether splenectomy predisposes one to increased or reduced risk of tumorigenesis and (2) the effect of splenectomy on tumor growth, progression, and relapse. For the first issue, epidemiological studies have observed that splenectomy is followed by increased risk for a large array of solid tumors and hematological malignancies (141–143). This finding is supported by a recent population-based cohort study demonstrating that people with splenectomy have an increased risk of developing overall cancer, as well as certain site-specific cancers, especially patients with non-traumatic conditions (144). These results suggest that the normal spleen plays an immune surveillance role, protecting against tumor development.

For the second issue, the effect of splenectomy pertaining to cancer progression has also been studied, but the evidence remains inconclusive. Studies on concomitant splenectomy in patients with gastric, colon, liver, and pancreatic cancers have shown marginal, if any, effects on the disease-free and overall survival of patients (145). Among these data, it may be of particular interest to look at the results from liver cancer because the so-called “liver-spleen axis” in liver disease is now gaining increasing attention (146–149). Liver transplantation (LT) has been established as a standard treatment for patients with HCC who meet the Milan criteria. Splenectomized LT patients benefit from increased platelet counts, but they suffer risks, including increased operation time, intraoperative blood loss, intraoperative red blood cell transfusion, and postoperative complications (150). Splenectomy improves patient prognosis but only in a subgroup of patients with an increased neutrophil-lymphocyte ratio (NLR) and increased infiltration of CD163^+^ TAMs in the tumor stroma, both of which are indicative of enhanced myelopoiesis (151). However, whether the abolishment of splenic myelopoiesis is directly involved in the therapeutic effect of splenectomy and the mechanisms by which splenic EMH, or lack thereof, may influence cancer progression and treatment are yet to be elucidated.

## Targeting Cancer-Induced Splenic Myelopoiesis

One explanation for the modest effect of splenectomy on tumor progression in both patients and mice is that the spleen is a multifunctional organ. As noted before, the spleen is an important organ for blood homeostasis and is a reservoir of various immune and blood cell populations that have differential impacts on tumor progression via diverse mechanisms. The ultimate impact of splenectomy on cancer patients is determined by the net balance of these known or still unknown factors, dependent on the individual's status. Therefore, an enhanced strategy is to seek a selective treatment modality that specifically targets protumoral splenic EMH while maintaining the normal physiological and antitumoral immune functions of the spleen (Figure 2).

In this context, Ugel et al. evaluated a large panel of conventional chemotherapeutic agents for their ability to eliminate splenic committed myeloid precursors. Low-dose 5-fluorouracil (5-FU) treatment, for example, could reduce the splenic (but not BM) expansion of committed precursors with high proliferative potential, restore antitumor immunity, and enhance the efficacy of ACT, recapitulating the effect of splenectomy (38). We recently found that a low-dose c-Kit inhibitor inhibits proliferation, induces apoptosis, and thus reduces the total number of upstream early HSPCs in the spleen but has a much smaller effect on those in the BM. Moreover, low-dose c-Kit inhibitor treatment attenuates endogenous GM-CSF expression in splenic HSPCs, inhibits the suppressive functions of tumor PMN-MDSCs, and synergistically increases the efficacy of immune checkpoint blockade (37). Why splenic HSPCs and committed myeloid precursors are more sensitive than their BM counterparts to such treatments is presently unclear. One possibility might be due to the anatomical structure and physiological function of the spleen, which often causes drug retention. Another possibility for the differential effects could be the distinct cellular characteristics of the BM and splenic HSPCs in tumor-bearing hosts. If so, a better understanding of the biological features of splenic HSPCs and myeloid precursors may provide a molecular basis for the development of novel therapeutic strategies to selectively target splenic myelopoiesis.

In addition to the regulation of splenic HSPC proliferation and survival, the specific abrogation of cancer-induced myelopoiesis could also be achieved by targeting the recruitment and retention of splenic HSPCs. In this scenario, the CCL2/CCR2 axis is attracting particular interest and plays multiple important roles in systemic tumor-associated myeloid cell responses. This axis mediates the migration of BM monocytes into the bloodstream (152), guides monocytes to the marginal zone of the spleen (38), and directs the infiltration of monocytes in the tumor (34, 153, 154). Moreover, as noted before, CCR2 expression identifies an upstream subset of circulating HSPCs that can respond to splenic CCL2 and home to the splenic niche (38). Thus, CCR2-specific antagonists may act as multivalent inhibitors targeting multiple events of cancer-induced myeloid cell responses. Currently, a number of clinical trials have been established to investigate the safety and efficacy of CCR2 inhibitors, including CCX872-B, PF-04136309, MLN1202, and BMS-813160, for the treatment of solid tumors [reviewed in (155)]. In addition, CD47 and AngII have been revealed as critical mediators of splenic HSPC retention and expansion. Blocking these signaling pathways may inhibit tumor-promoting splenic myelopoiesis, as shown in mouse models (75, 122). Nevertheless, the translational values of these findings need to be further investigated in cancer patients to validate whether the blockade of these signals will be effective and beneficial and, importantly, whether the therapeutic effects rely on the impact on altered myelopoiesis in the spleen.

## Concluding Remarks and Future Perspectives

The emerging field of cancer-induced hematopoiesis, EMH in particular, complements and completes our knowledge of tumor-associated myeloid responses. The spleen, as the main EMH site in tumor-bearing hosts, generates significant amounts of myeloid cells that continuously replenish the large and rapidly turned over pool but is functionally and mechanistically different from that in the BM. The understanding of the unique splenic myelopoiesis opens a new avenue of myeloid cell-targeting strategies, which pursue the goal of restraining systemic tumor-promoting myeloid responses at their source.

From the therapeutic perspective, splenic myelopoiesis may be the “weakest link” in the chain of myeloid cell reactions because the spleen is a rather pharmacodynamically favorable organ due to its anatomical structure and the large blood flow (75). In addition, splenic HSPCs, partially due to their highly proliferative nature and residence in a less protective niche, are more vulnerable to targeted drugs than their BM counterparts and downstream myeloid descendants (37, 38). Therefore, targeting splenic myelopoiesis holds real potential to restrain tumor-promoting myeloid cell responses and to tip the balance toward tumor suppression. A better understanding of the functional specialization and regulatory mechanism of splenic myelopoiesis will provide the keys to controlling myeloid cell responses at the source.

Finally, more human data are needed to demonstrate the clinical relevance of splenic myelopoiesis in cancer patients. Studies on cancer-induced splenic myelopoiesis in humans are hampered by the limited availability of spleen samples, the poorly defined phenotypes and functions of the highly heterogeneous circulating HSPC subsets, and the unclear nature of the splenic niche constitution. *In situ* studies using novel multiplex staining and detection methods, lineage-tracing and imaging techniques, and informative tools and statistical modeling would be invaluable for identifying disease-specific splenic myelopoiesis patterns. Single-cell analyses, such as cytometry by time of flight (CyTOF) and single-cell RNA sequencing, can help to reveal the heterogeneity of splenic HSPC populations in different conditions. Dynamic modeling using *in vitro* experiments will be crucial to identify key regulatory pathways and search for checkpoints that are susceptible to therapy. These advanced methodologies and experimental models will not only facilitate human studies but also facilitate the translation of clinical insights back to improvements in mouse models, which may produce applicable and precise therapeutics. Such parallel studies may provide a long sought-after means to reshape the tumor immune micro- and macroenvironment by rerouting myeloid cell responses.

## Author Contributions

All authors listed have made a substantial, direct and intellectual contribution to the work, and approved it for publication.

## Conflict of Interest

The authors declare that the research was conducted in the absence of any commercial or financial relationships that could be construed as a potential conflict of interest.
